# Anti-inflammatory effects of astroglial α7 nicotinic acetylcholine receptors are mediated by inhibition of the NF-κB pathway and activation of the Nrf2 pathway

**DOI:** 10.1186/s12974-017-0967-6

**Published:** 2017-09-26

**Authors:** Hiral Patel, Jessica McIntire, Sarah Ryan, Anthone Dunah, Ralph Loring

**Affiliations:** 1 Department of Pharmaceutical Sciences, Northeastern University, Boston, MA USA; 2 Neurology Research, Biogen, 225 Binney street, Cambridge, MA 02142 USA; 3 Pre-Clinical Imaging & Pharmacology, Biogen, Cambridge, MA USA

**Keywords:** α7 nicotinic acetylcholine receptors, Astrocytes, NF-κB pathway, Nrf2 pathway

## Abstract

**Background:**

α7 nicotinic acetylcholine receptors (nAChRs) are widely distributed throughout the central nervous system and are reported to have neuroprotective properties. α7 nAChRs are expressed on astrocytes, which are key regulators of neuroinflammation and oxidative stress in several neurodegenerative diseases. However, the anti-inflammatory and antioxidant properties of astroglial α7 nAChRs are not well studied. Therefore, we evaluated the role of astroglial α7 nAChR activation in neuroinflammation.

**Methods:**

Anti-inflammatory and antioxidant effects of α7 nAChR activation were evaluated in an in vitro mouse model of neuroinflammation using lipopolysaccharide (LPS) in primary astrocyte cultures. α7 nAChR anti-inflammatory effects on the NF-κB pathway were evaluated using ELISA, gene expression analysis, immunofluorescence, and western blotting. Antioxidant effect of α7 nAChR activation on expression profiles of canonical Nrf2 target genes was examined by quantitative PCR and western blotting. The role of the Nrf2 pathway in α7 nAChR-mediated anti-inflammatory response was evaluated using Nrf2 knockout astrocytes. Brain ex vivo NF-κB luciferase signals were evaluated after treatment with an α7 nAChR agonist in lipopolysaccharide (LPS)-injected NF-κB luciferase reporter mouse model.

**Results:**

Astrocytes treated with the α7 nAChR partial agonist (GTS21) showed significantly reduced LPS-mediated secretion of inflammatory cytokines and this effect was reversed by the α7 nAChR antagonist methyllycaconitine (MLA) and by knockdown of α7 nAChR expression with a short hairpin RNA. Further, α7 nAChR activation blocked LPS-mediated NF-κB nuclear translocation indicating that the observed anti-inflammatory effect may be mediated through inhibition of the NF-κB pathway. Treatment with GTS21 also upregulated canonical Nrf2 antioxidant genes and proteins suggesting antioxidant properties of α7 nAChR in astrocytes. Using an astrocyte conditioned media approach, we demonstrated reduction in neuronal apoptosis when astrocytes were pretreated with GTS21. Finally, in an in vivo neuroinflammation model using LPS in NF-κB luciferase reporter mice, we demonstrated reduction in LPS-induced NF-κB activity and pro-inflammatory cytokines with GTS21 treatment in brain tissue.

**Conclusion:**

Our results suggest that activating astroglial α7 nAChRs may have a role in neuroprotection by decreasing inflammation and oxidative stress, and therefore could have therapeutic implication for disease modifying treatments of neurodegenerative diseases.

## Background

α7 nicotinic acetylcholine receptors (nAChRs) are widely distributed throughout the central nervous system (CNS) and periphery [1]. Within the CNS, these receptors are expressed in neurons and glial cells and are actively involved in learning, memory, and attention [2]. Observations from neuronal cell lines, primary neuron cultures, and transgenic mice with deleted α7 nAChR indicate that agonists of these receptors, including galantamine (an allosteric modulator), PNU-282987, TC-1698, and GTS21 (3-[2,4-dimethoxybenzylidene]anabaseine), provide neuroprotection against toxicity induced by various insults such as amyloid-beta, glutamate, okadaic acid, and ethanol which are reversed by α7 nAChR antagonists such as methyllycaconitine (MLA) or α-bungarotoxin [3–10]. Therefore, α7 nAChRs are of great interest as potential therapeutic target in various neurodegenerative diseases.

While the neuroprotective role of α7 nAChRs is well characterized, limited evidence exists regarding the potential anti-inflammatory properties of these receptors in astrocytes. Functional α7 nAChRs are reported to be present on astrocytes, which upon activation increase intracellular calcium levels [11]. Some preliminary data point towards potential anti-inflammatory effects mediated through α7 nAChRs expressed in astrocytes. Liu et al. demonstrated that activation of astroglial α7 nAChRs may provide protection against degeneration of dopaminergic neurons by inhibition of MPTP (in vivo)- and MPP+- or LPS (in vitro)-induced astrogliosis in Parkinson’s disease [12]. However, the molecular mechanism of the observed anti-inflammatory response of α7 nAChR and resultant neuroprotection has not been studied. In non-neuronal cells such as monocytes and macrophages, anti-inflammatory properties of α7 nAChRs are reported to be mediated through inhibition of the NF-κB pathway [13, 14]. More recently, it has been demonstrated that activation of α7 nAChRs in glial cells leads to blocking of the NF-κB pathway and a consequent reduction in neuroinflammation [15]. Another emerging mechanism that may explain α7 nAChR-mediated neuroprotection is the Nrf2 pathway. Nrf2 is a member of the NF-E2 family of basic region leucine-zipper transcription factors and responds to oxidative and electrophilic stress by regulating antioxidant responsive genes. Recent evidence from rat organotypic hippocampal slice culture suggests that α7 nAChR agonists induce heme oxygenase 1 via the Nrf2 pathway in a brain ischemic model, which provides neuroprotection [16].

Evaluating the potential role of astroglial α7 nAChRs is critical because astrocytes are the most abundant cell type in the brain, representing 20–40% of the brain cells. Astrocytes are increasingly being recognized as important mediators of neuroinflammation and consequent cognitive impairment [17, 18]. Therefore, targeting astroglial α7 nAChRs for their neuroprotective properties may be important in guiding development of disease modifying treatments for various neurodegenerative diseases. Thus, we used in vitro and in vivo models of neuroinflammation to elucidate the molecular mechanism for anti-inflammatory and antioxidant properties of α7 nAChRs activation in astrocytes, based on the hypothesis that the crosstalk between the NF-κB and Nrf2 pathways mediates these effects.

## Methods

### Cell culture

Astrocytes were purified from cortices of postnatal day 2 C57BL/6 mouse pups using a shaking method described by McCarthy and de Villis [19]. Briefly, we first dissected the cortical tissues and then removed the meninges. The tissues were then centrifuged and washed with cold Hank’s Balanced Salt Solution (HBSS). Tissue dissociation was performed using Papain-based Neural Tissue Dissociation Kit (Miltenyi Biotec, Inc.).Tissues were further incubated with DNAse and mixed until well dissociated. After dissociation, cells were filtered, centrifuged, and resuspended in DMEM supplemented with 20% fetal bovine serum (FBS), penicillin, and streptomycin. Finally, cells were counted and plated in poly-D-lysine coated T-75 cm^2^ flasks. Medium was replaced after 4–5 h and then every 3 days. After 8 days in culture, flasks were shaken on an orbital shaker for 18–20 h at 200 rpm to release microglia and oligodendrocytes from the mixed cultures. Purified astrocytes were then trypsinized and replated for assay.

Neurons were isolated from embryonic day 16–18 mouse brains. Brains were first collected in a 100-cm petri dish filled with cold HBSS. Next, the brainstem and meninges were removed using a dissecting microscope. Then, cortices were dissected and collected in 15 ml conical tube with HBSS. Cortical tissues were washed with cold sterile HBSS twice followed by addition of trypsin. Tissues were incubated with trypsin at 37 °C for 20 min. After incubation, DNAse I was added to tissue for 30 s. To meet the special cell culture requirements of pre-natal and embryonic neuronal cells, dissociated cells were filtered and plated in neurobasal medium (Gibco, catalog no. 21103, with 2% B27 (Gibco, catalog no. 17504-044), 1% glutamax (Gibco, catalog no. 35050), and 10 units/ml penicillin streptomycin (Gibco, catalog no. 15140-148).

### Astrocyte treatment paradigm

Astrocytes were plated in 24 well plates at the density of 100,000 cells/well in DMEM with 20% FBS medium for 24 h. Cells were serum starved before adding compounds. Cells were then stimulated with or without 60 ng/ml LPS (Sigma, L6529) in the presence or absence of different doses of GTS21 (Sigma) to measure cytokine levels.

### α7 nAchR gene knockdown with short hairpin RNA

Astrocytes were transduced with either lenti short hairpin (sh)-RNA for α7 nAchR or scrambled shRNA in 6-well plate for 7 days. Two different sequences of 29mer α7 nAchR-specific shRNA were used (GCCAACGACTCGCAGCCGCTCACCGTGTA, CTTGGAATAACTGTCTTACTTTCTCTGAC) to maximize knockdown efficiency. Knockdown efficiency was determined using qRT-PCR with TaqMan probes specific for α7 nAchR (Assay ID; Mm01312230_m1). Next, the cells were treated with LPS (60 ng/ml) in the presence or absence of 30 μm GTS-21. After treatment, RNA was isolated from each condition and TNF levels were measured.

### Measurement of secreted TNF-α and IL-6 using enzyme-linked immunosorbent assay (ELISA)

TNF-α secretion was measured in purified astrocyte cultures treated with LPS in the presence or absence of GTS21. GTS21 pretreatment was done for 1 h followed by 24 h of LPS treatment. Following the treatment, cell-free culture medium was collected and TNF-α and IL-6 were measured using a commercially available mouse colorimetric ELISA assay (eBiosciences) following the manufacturer’s protocol.

### Immunocytochemistry

For immunofluorescence, purified astrocytes were plated for 24 h and fixed with 4% paraformaldehyde for 15 min and washed three times with PBS. After fixation, cells were permeablized with 1× GDB buffer (Gelatin Solution, 0.3% Triton X-100, Phosphate Buffer, NaCl). Cells were then incubated with anti-GFAP antibody (Sigma, G6171) and anti- NF-κB antibody (Abcam, ab16502) overnight at 4 °C, followed by washing three times with PBS and 1 h incubation of Alexa-488 or 594 conjugated secondary antibody (Thermofisher). DAPI (Thermofisher) was used as nuclear stain.

For α-bungarotoxin labeling, primary astrocytes were plated in 96-well plate at 20,000 cells per well for 24 h. Thereafter, cells were pretreated with 200 μm nicotine for 15 min followed by treatment with Alexa Fluor 488-conjugated α-bungarotoxin (B13422, Thermofisher Scientific) at 4 °C for 15 min. Immediately following incubation, these cells were washed with PBS three times and then fixed for 15 min in 4% paraformaldehyde at room temperature. After fixation, cells were washed twice with PBS and examined under a fluorescent microscope.

### Immunostaining quantitation

Images were captured and analyzed using Cellomics ArrayScan XTI high-content analysis system. This system contains high-resolution photometrics X1 CCD, 14 bit camera used for automated image acquisition. The Cellomics Target Activation bioapplication was used for processing and analyzing the images to quantitate the percentage purity of astrocytes within a well, and the spot detection bioapplication was used to quantitate the percentage of NF-κB nuclear translocation and caspase 3/7 fluorescence within a well.

### Total RNA extraction from cells

Total RNA was extracted from astrocytes using RNeasy miniprep plus kit from Qiagen according to the manufacturer’s instructions. Genomic DNA was eliminated using the genomic DNA eliminator column provided with the RNA isolation kit. Purified RNA was quantified using a NanoDrop® ND-1000 UV-Vis Spectrophotometer. The quality of RNA was determined by using OD 260/OD 280 and OD 260/OD 230 which were approximately 1.8–2. Total RNA was reverse transcribed into DNA using a high capacity cDNA reverse transcription kit (Applied Biosystems) using the manufacturer’s protocol.

### Total RNA extraction from tissues

RNA extraction from frozen tissues was performed using QIAzol extraction method. Frozen tissues were collected in an RNase free deep-well plate (96-well plate from Corning, 3961). A 2.3 mm stainless steel bead was added to each of the frozen tissues. Tissues were then disrupted by adding QIAzol reagent followed by subjecting the block to Mini-Beadbeater for 4 cycles of 45 s each. This resulted in efficient disruption of the tissues. The aqueous layer was collected after mixing with chloroform. An equal volume of 70% ethanol was added to the aqueous layer, mixed thoroughly, and applied to RNeasy 96 plates (Qiagen). Purification of RNA was done according to the manufacturer’s protocol.

### Real-time polymerase chain reaction (rt-PCR)

Target gene primers along with 6-FAM™ dye-labeled TaqMan micro groove binder probe for quantitative PCR analysis were obtained from Applied Biosystems (assay IDs Mm00516005_m1 for heme oxygenase 1 (HO1), Mm00443675_m1 for thioredoxin reductase (TXNRD1), Mm00802655_m1 glutamate-cystein ligase catalytic subunit (GCLC), and Mm00660947_m1 for oxidative stress-induced growth inhibitor (OSGIN1)). Each reaction contained 100 ng of DNA, 900 nM each of forward and reverse primers, and 250 nM TaqMan probe. Temperature conditions consisted of a 10 min cycle at 95 °C, followed by 40 cycles of 95 °C for 0.15 min and 60 °C for 1 min using Stratagene Mx 3005P. All samples were measured in at least duplicates along with GAPDH as a normalizing gene, and the no-template control was negative for all runs. The final analysis was done using comparative CT method.

### Gene expression analysis

The TaqMan® OpenArray® Gene Expression platform was used to perform a transcriptomic analysis to comprehensively evaluate inflammatory responses upon α7 nAchR activation in an in vitro inflammation model using LPS. Total RNA extracted from astrocytes treated with LPS alone and LPS in the presence of GTS21 was reverse transcribed into cDNA. The cDNA was then loaded to a TaqMan® OpenArray® Mouse Inflammation Panel plate (Thermofisher, 4475393) consisting of 632 gene targets selected for their involvement in inflammatory responses. The QuantStudio 12K Flex Real-Time PCR System (Thermofisher) was used to perform real-time PCR and quantitative gene expression. Fold changes (RQ) in expression of inflammatory genes were calculated using comparative CT method. A corrected *p* value of 0.05 was used to identify differentially expressed genes.

Next, based on these differentially expressed genes, a gene set enrichment analysis was conducted using the software package Generally Applicable Gene-set Enrichment (GAGE) in R Bioconductor [20]. This software provides a list of Kyoto Encyclopedia of Genes and Genomes (KEGG) mouse pathways that are enriched by the differentially expressed genes. We aimed to identify pathways that are significantly downregulated upon treatment with GTS21. The R package, Pathview, was used to visualize maximally enriched KEGG pathways.

### Nitrite assay

We used the Griess reagent (Promega) to evaluate the production of nitrite (NO_2_^−^), which is a breakdown product of nitric oxide (NO). Astrocytes were pretreated with GTS21 followed by LPS treatment for 3 days in DMEM in the absence of phenol red. After the treatment, cell-free conditioned medium was collected for the assay. First, the medium was incubated with reagent A containing sulfanilamide followed by incubation with reagent B, N-1-napthylethylenediamine dihydrochloride. This resulted in the formation of colored compound, which was measured at 540 nm using a microplate reader.

### Neuronal survival assay

For this assay, we added LPS-treated astrocyte conditioned medium in the presence or absence of α7 nAchR agonist GTS21, in cultured neurons for 24 h. Caspase-3 activation was measured using The CellEvent® Caspase-3/7 green detection reagent (Thermofisher). Images were captured and analyzed using Cellomics ArrayScan XTI high-content analysis system, and spot detection bioapplication was used for processing and analyzing the images to quantitate the percentage of caspase-3 activation within a well.

### Protein isolation and western blot analysis

Cells were scraped in 1× lysis buffer from Cell Signaling Technology (catalog no. 9803) with protease and phosphatase inhibitors and 0.5% SDS, followed by sonication with F60 Sonic Dismembrator (Fisher Scientific). The amount of protein in each sample was quantified using Pierce BCA Protein Assay Kit. All samples were diluted to the same concentration. For western blot analysis, samples were boiled at 95 °C for 5 min and 20 μg of total protein was loaded on Criterion™ TGX™ precast gels (BioRad Laboratories). Gels were run for 1 h at 150 V followed by transferring proteins onto a nitrocellulose membrane using an iBlot system (Invitrogen). Five percent non-fat dried milk in tris-buffered saline, tween 20 was used to block the membranes for 1 h. After blocking, primary antibodies against GCLC (Abcam), NQO1 (Abcam), HO1 (Abcam), TXNRD1 (Abcam), ACTB (MP Biomedical), Phospho-IκBα (Abcam), and GAPDH (Abcam) were incubated overnight at 4 °C, followed by washing with 1× tris-buffered saline, tween 20 for 30 min. Secondary antibodies were then incubated at room temperature for 45 min. After washing the membranes for 30 min, Super Signal West Femto Substrate was added to detect horseradish peroxidase on the membranes. Chemiluminescence was measured and quantified using the Syngene gel imaging system.

### Animals

All procedures involving animals were approved by the Biogen Institutional Animal Care and Use Committee, which is accredited by the Association for Assessment and Accreditation of Laboratory Animal Care International. Eight- to 12-week-old NF-κB luciferase reporter mice were used for in vivo experiments [21]. Four groups of five mice were used. In the first group, mice were administered with phosphate buffer saline (PBS) intraperitoneally twice; in the second group, mice were first administered with PBS followed by LPS (1.7 mg/kg) intraperitoneally; in the third group, mice were first administered with GTS21 (5 mg/kg) followed by LPS (1.7 mg/kg) intraperitoneally; and in the fourth group, mice were first administered with GTS21 (25 mg/kg) followed by LPS (1.7 mg/kg) intraperitoneally. At 4 h, mice were euthanized using CO_2_ asphyxiation method for ex vivo brain imaging and tissue collection.

### NF-κB luciferase brain ex vivo imaging

Brain ex vivo NF-κB luciferase signals were evaluated in images acquired on the IVIS Spectrum instrument (Perkin Elmer, Hopkinton, MA), using the Perkin Elmer proprietary software, Living Image (v4.3.1). Luciferin was injected intraperitoneally at 150 mg/kg for ex vivo brain imaging, which was conducted after euthanizing the animal and harvesting the brain. One hemisphere of the brain was imaged, and the other half was collected for RNA analysis.

### Data analysis

Experimental results were analyzed using GraphPad Prism software. Data are expressed as mean (+/− standard deviation). Differences in means for continuous dependent variables were compared using unpaired Student *t* tests (for two groups) or one-way ANOVA with adjustment for multiple comparisons (for more than two groups). Tukey’s test was used for multiple comparisons when considering all pairwise comparisons and Dunnett’s test was used when comparing multiple groups to a common control. Two-tailed *p* values of less than 0.05 were considered as statistically significant.

## Results

### LPS triggers release of pro-inflammatory cytokines, NF-κB nuclear translocation, and morphological changes in astrocytes

**Fig. 1:**
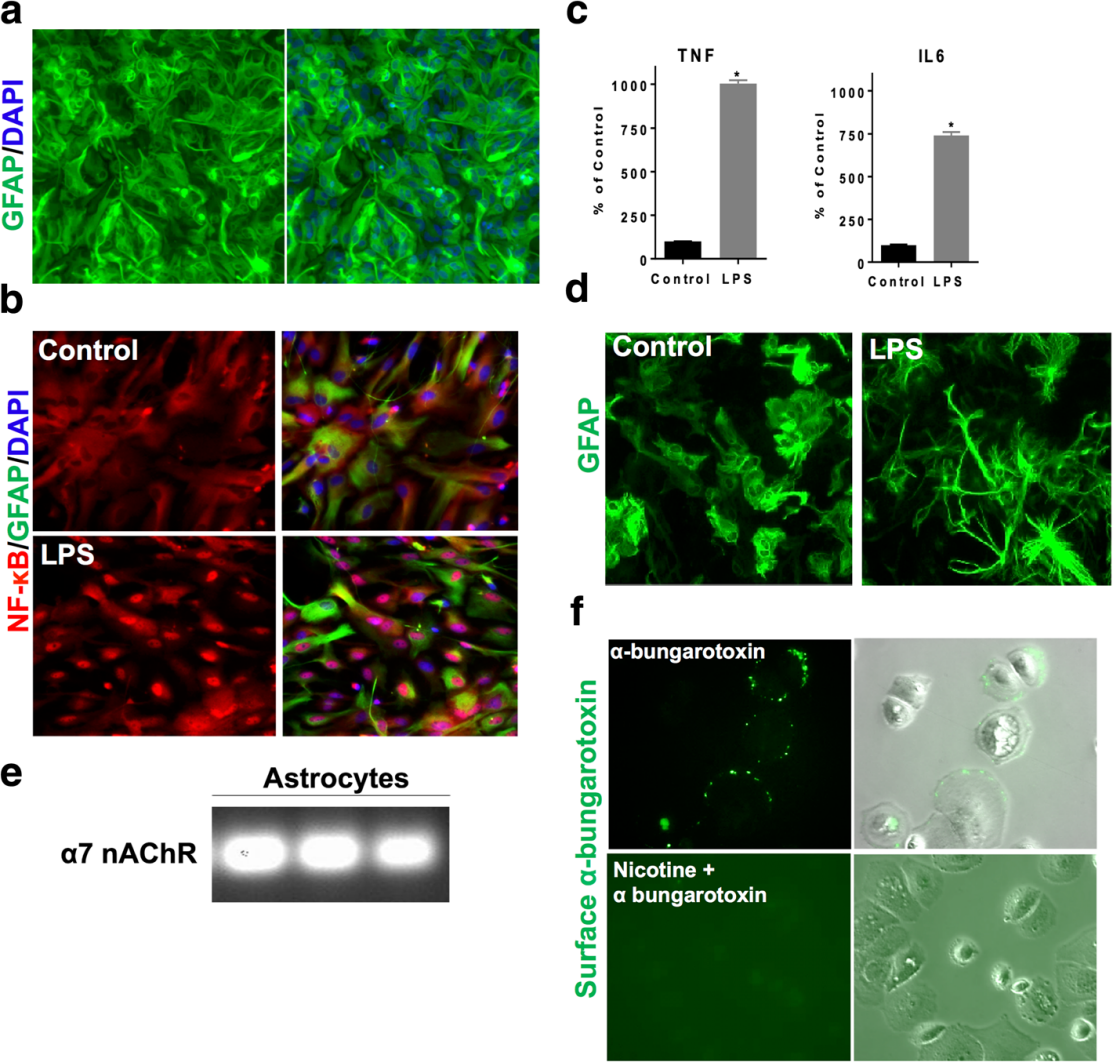
Characterization of an in vitro model of neuroinflammation and expression of α7 nAchRs in mouse astrocytes. **a** These are representative images of mouse astrocytes using the Cellomics ArrayScan XTI high-content analysis system for automated image acquisition of 20 fields for each well at × 20 magnification. In each field, two channels were captured to image nuclei (DAPI) and astrocyte marker (GFAP). The ArrayScan Target Activation bioapplication was used for processing and analyzing the images to quantify GFAP positive cells as percentage of total cell counts using DAPI within a well. This was found to be more than 90%. **b** LPS (60 ng/ml) treatment for 3 h increased nuclear translocation of p65 subunit of NF-κB in astrocytes as a marker of acute inflammation phase. **c** LPS (60 ng/ml) treatment for 24 h resulted in significant increase in pro-inflammatory cytokines, TNF and IL-6, downstream of NF-κB activation in astrocytes, *p < 0.05 as compared with untreated (*t* test). Error bars represent SD (*n* = 4). **d** Stimulation of astrocytes with LPS (60 ng/ml) for 24 h increased cell processes substantially as compared to the control cells. **e** α7 nAChR subunit mRNA expression observed in cultured astrocytes (three replicates). **f** Binding of fluorescently labeled α-bungarotoxin was observed in astrocytes. Pretreatment with 200 μm nicotine (α7 nAchR agonist), significantly inhibited binding of fluorescently labeled α-bungarotoxin shown by decrease in fluorescence confirming expression of α7 nAchR in these cells

The purity of astrocyte culture was measured by immunofluorescence by astrocyte cell specific marker, glial fibrillary acidic protein (GFAP). Using the ArrayScan XTI high-content analysis system, the purity was determined to be greater than 90% based on GFAP positive cells as a proportion of the total cell number (Fig. 1a). LPS is known to stimulate inflammatory responses in a variety of cells including astrocytes [12, 22]. Therefore, we used LPS as a stimulus to activate astrocytes and characterize their inflammatory response. We characterized the standard astrogliosis response upon LPS treatment. As indicated in Fig. 1b, immunofluorescence showed a significant increase in NF-κB nuclear translocation after 3 h of LPS treatment as compared to untreated cells. As shown in Fig. 1c, we observed a statistically significant increase in pro-inflammatory cytokines TNF-α and IL-6 measured using ELISA after 24 h of treatment with LPS. LPS also modified the morphology of astrocytes by increasing cell processes as compared to the control cells (Fig. 1d).

### α7 nAchRs are expressed in mouse astrocytes

To evaluate the expression of α7 nAChRs in primary astrocyte cultures, we performed quantitative RT-PCR using TaqMan probes specific for α7 nAChR (Applied Biosystem (ThermoFisher Scientific) assay ID Mm01312230_m1). As shown in Fig. 1e, our results confirm α7 nAChR subunit mRNA expression in cultured astrocytes. There was no genomic DNA detected in these samples (data not shown). Next, we evaluated surface binding of an impermeable competitive α7 nAChR antagonist, α-bungarotoxin, to further confirm their expression on astrocytes. Upon treatment of astrocytes with Alexa fluor-488-labeled α-bungarotoxin, we observed surface binding as shown in Fig. 1f. Pretreatment with nicotine significantly inhibited binding of fluorescently labeled α-bungarotoxin shown by a decrease in fluorescence. Reduction in surface biding of α-bungarotoxin, which is a competitive α7 nAChR antagonist, in the presence of an agonist (nicotine) in our astrocyte cultures further confirms expression of α7 nAChRs on astrocytes.

### α7 nAChR activation reduces LPS-mediated pro-inflammatory cytokine secretion in astrocytes

**Fig. 2:**
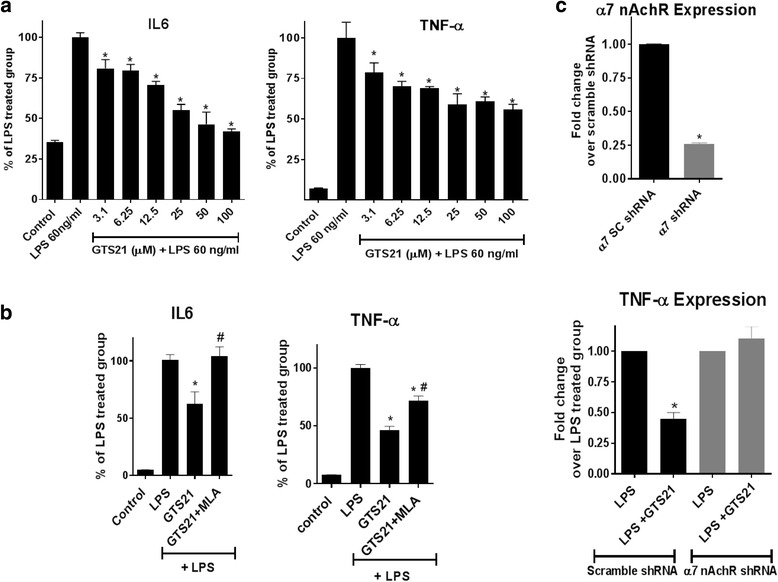
α7 nAchR activation reduced LPS-mediated pro-inflammatory cytokine secretion in astrocytes. **a** Pretreatment with GTS21 (α7 nAchR agonist) for 1 h resulted in a dose-dependent reduction in both IL-6 and TNF-α secretion as compared to LPS treated alone for 24 h, *p < 0.05 compared with LPS (60 ng/ml) treatment (one-way ANOVA with adjustment for multiple comparisons using Dunnett’s test). Error bars represent SD (*n* = 4). **b** Pretreating the cultured astrocytes for 1 h with 1 μm α7 nAchR antagonist (MLA) resulted in blockage of anti-inflammatory response of 20 μm GTS21, as measured by secretion of pro-inflammatory cytokines TNF-α and IL-6 in astrocytes treated with LPS for 24 h. *p < 0.05 compared with LPS 60 ng/ml treatment, ^#^ *p* < 0.05 compared to respective agonist treatment alone (both using one-way ANOVA with adjustment for multiple comparisons using Tukey’s test). Error bars represent SD (*n* = 4). **c** Knocking down α7 nAchR expression with lenti short hairpin RNA in astrocytes resulted ~ 75% gene knockdown, which significantly blocked the anti-inflammatory response of GTS21 (30 μm) in astrocytes treated with LPS for 24 h. Error bars represent SD (*n* = 2)

To investigate the effect of α7 nAchR activation on the secretion of pro-inflammatory cytokines TNF-α and IL-6, we pretreated astrocytes with α7 nAChR agonist GTS21 for 1 h followed by 24 h of LPS treatment. To ascertain the dose-response effect of α7 nAchR activation on the secretion of TNF-α and IL-6, we used increasing doses of GTS21 (from 3 to 100 μm). LPS caused significant increase in IL-6 and TNF-α secretion in these cells and GTS21 pretreatment resulted in a dose-dependent reduction in LPS-mediated IL-6 and TNF-α secretion (Fig. 2a). No difference in the cell viability was observed due to treatments (data not shown).

To ascertain whether the anti-inflammatory effects observed by GTS21 are specific to α7 nAChRs, GTS21 treatment was done in the presence or absence of the α7 nAChR-specific antagonist, methyllycaconitine (MLA). Following these treatments, cells were activated with LPS and TNF-α and IL-6 were measured using ELISA in the cell-free supernatant. As demonstrated in Fig. 2b, we observed that treatment with MLA significantly reversed the blockade of TNF-α and IL-6 secretion by GTS21. We also transduced astrocytes with either lenti short hairpin RNA for α7 nAChR or scrambled shRNA and observed approximately 75% knockdown using qRT-PCR with TaqMan probes specific for α7 nAchR (Fig. 2c). Upon knockdown of α7 nAChR in astrocytes, GTS21 was unable to reduce the levels of LPS-mediated TNF-α expression (Fig. 2c).

### α7 nAChR activation results in inhibition of the NF-κB signaling pathway

**Fig. 3:**
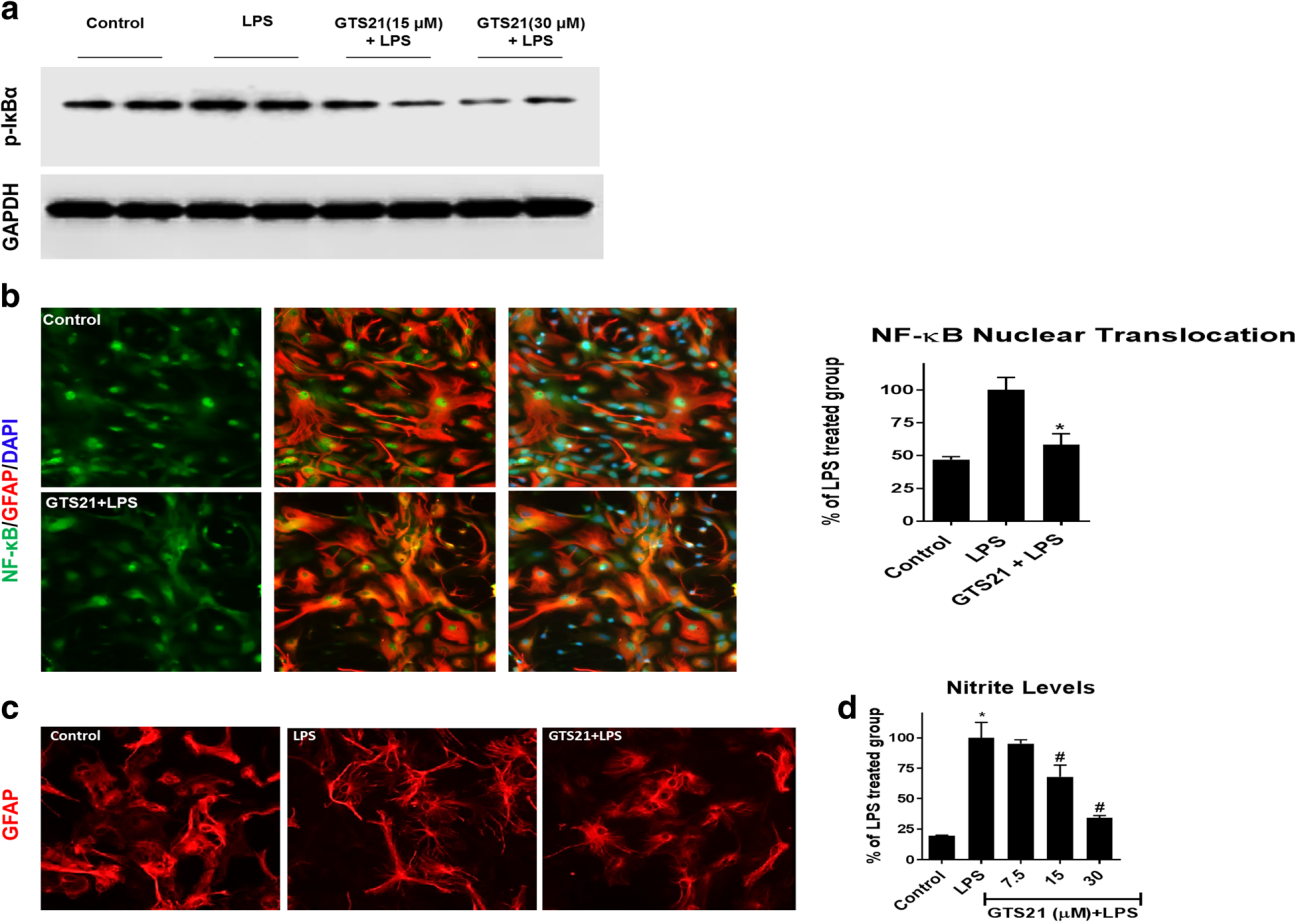
α7 nAchR activation resulted in inhibition of the NF-κB signaling pathway and astrogliosis. **a** Pretreatment with GTS21 (15 and 30 μm) resulted in reduction of phosphorylated form of IκBα in LPS (60 ng/ml)-treated astrocytes for 30 min. **b** Treatment of astrocytes with LPS (60 ng/ml) for 3 h caused robust nuclear translocation of NF-κB and pretreatment with α7 nAchR agonist GTS21 (30 μm) for 1 h caused significant reduction in NF-κB nuclear translocation, *p < 0.05 (one-way ANOVA with adjustment for multiple comparisons using Dunnett’s test for the comparison of LPS alone group and GTS21+LPS group as percentage of LPS alone group). Error bars represent SD (*n* = 6 wells, 50 fields/well). **c** Treatment with GTS21 (30 μm) reduced the number of processes in LPS (60 ng/ml for 24 h)-activated astrocytes suggesting reduction in reactive astrogliosis. **d** Pretreatment of GTS21 with 7.5, 15, and 30 μm for 1 h resulted in a dose-dependent decrease in LPS (60 ng/ml for 72 h)-induced levels of nitrites further confirming anti-inflammatory properties of α7 nAchR activation. *p < 0.05 compared with control, ^#^ *p* < 0.05 compared with LPS, 60 ng/ml treatment (both using one-way ANOVA with adjustment for multiple comparisons using Tukey’s test). Error bars represent SD (*n* = 6)

To investigate the mechanism underlying the anti-inflammatory properties of GTS21 in LPS-stimulated astrocytes, we evaluated the effect of GTS21 on the NF-κB pathway. For this experiment, we evaluated different components of this pathway in astrocytes stimulated with LPS in the presence or absence of GTS21 beginning from early events (IκBα phosphorylation and NF-κB nuclear translocation) to later events (reactive astrogliosis and nitrite production). First, we evaluated the effect of α7 nAChR activation on IκBα, which binds to NF-κB and prevents its nuclear translocation. Phosphorylation of IκBα indicates its inactivation [23, 24]. Astrocytes were pretreated with GTS21, followed by treatment with LPS for 30 min since this event occurs earlier in the NF-κB signaling cascade upon LPS stimulation. Following treatment, the cells were lysed and the phosphorylated form of IκBα was measured. LPS treatment resulted in increased levels of the phosphorylated form of IκBα, indicating nuclear translocation and activation of the NF-κB pathway. However, pretreatment with GTS21 resulted in a reduction of phosphorylated IκBα (Fig. 3a). Next, we evaluted nuclear translocation of NF-κB using immunofluorescence with the ArrayScan XTI high-content analysis system. As shown in Fig. 3b, astrocytes treated with LPS for 3 h showed robust NF-κB nuclear translocation, but the α7 nAchR agonist GTS21 significantly decreased this effect. We then evaluated LPS-mediated reactive astrogliosis by monitoring morphological changes in astrocytes upon α7 nAchR activation. Astrocytes were pretreated with GTS21 and stimulated using LPS for 24 h. We observed that treatment with LPS modified the morphology of astrocytes by increasing cell processes and branches. However, GTS21 pretreatment reduced the number of processes in activated astrocytes, suggesting a reduction in reactive astrogliosis (Fig. 3c). Finally, we measured nitrite levels, a breakdown product of the pro-inflammatory signaling molecule nitric oxide, using the Griess reaction. With LPS treatment for 72 h, we observed a significant increase in nitrite levels. However, a dose-dependent decrease in the levels of nitrite was noted with GTS21 pretreatment (Fig. 3d).

### α7 nAChR activation reduces LPS-mediated induction of inflammatory genes associated with the NF-κB signaling pathway

**Fig. 4:**
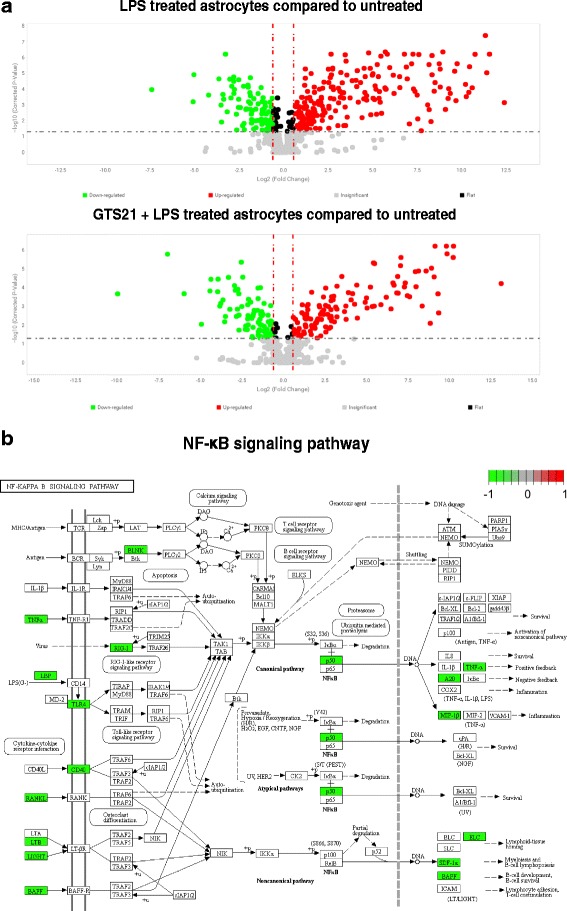
α7 nAchR activation reduced LPS-induced inflammatory genes in astrocytes. **a** LPS-mediated inflammatory genes were measured using TaqMan® OpenArray® Mouse Inflammation Panel covering 632 inflammatory genes, and changes were plotted on a volcano plot with log fold changes on *x*-axis and log of corrected *p* values on *y*-axis. Red vertical lines indicate boundaries for fold changes. Green dots indicate at least 1.5-fold lower expression and red dots indicate at least 1.5-fold higher expression (both statistically significant at *p* < 0.05). Gray dots, plotted below the horizontal line, indicate statistically non-significant changes. Pretreatment of astrocytes with GTS21 (30 μm) for 1 h followed by LPS (60 ng/ml) treatment for 24 h resulted in a substantially lower number of inflammatory genes upregulation (fewer red dots in the lower panel) indicating a robust anti-inflammatory effect of α7 nAchR activation. **b** A gene set enrichment analysis was performed using the software package Generally Applicable Gene-set Enrichment (GAGE) in R Bioconductor based on the 214 differentially expressed inflammatory genes with GTS21 treatment to identify KEGG pathways enriched by the downregulation of inflammatory genes in the GTS21 plus LPS-treated samples compared to LPS alone treated samples. This plot shows the NF-κB signaling pathway, which was statistically significantly downregulated with GTS21 treatment (*p* value 0.04), along with specific genes that were downregulated in green shaded boxes

Mouse astrocytes were treated with PBS alone, PBS in the presence of LPS or GTS21 in the presence of LPS for 24 h. Reverse-transcribed cDNA was loaded to a TaqMan® OpenArray® mouse inflammation panel covering 632 genes specifically targeted for inflammation. Figure 4a is a volcano plot demonstrating upregulation of inflammatory genes upon LPS treatment compared to PBS control. Pretreatment with GTS21 resulted in a substantially lower number of inflammatory genes upregulated as compared to LPS indicating a robust anti-inflammatory effect of α7 nAChR activation.

We then performed a gene set enrichment analysis using the software package Generally Applicable Gene-set Enrichment (GAGE) in R Bioconductor [20] based on the 214 differentially expressed inflammatory genes with GTS21 treatment. In this analysis, we identified the KEGG pathways enriched by the downregulation of inflammatory genes in the GTS21 plus LPS-treated samples compared to LPS alone treated samples. We observed that the NF-κB signaling pathway was statistically significantly downregulated with GTS21 treatment (*p* value 0.04, Fig. 4b).

### α7 nAChR activation induces upregulation of canonical Nrf2 antioxidant genes

**Fig. 5:**
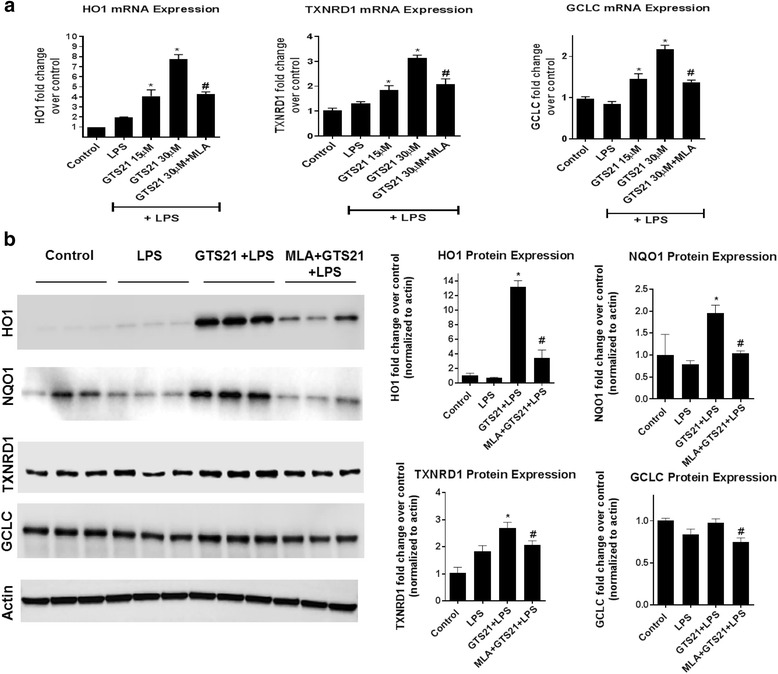
α7 nAchR activation induced upregulation of canonical Nrf2 antioxidant genes. **a** Pretreatment with GTS21 (15 and 30 μm) for 1 h resulted in significant upregulation of canonical Nrf2 antioxidant genes HO1, TXNRD1, and GCLC, in astrocytes treated with LPS for 24 h. α7 nAchR antagonist MLA (1 μm) significantly reduced this effect indicating the specificity of this response. *p < 0.05 compared with LPS, 60 ng/ml treatment, ^#^ *p* < 0.05 compared with GTS21 30 μm + LPS 60 ng/ml (both using one-way ANOVA with adjustment for multiple comparisons using Tukey’s test). Error bars represent SD (*n* = 4). **b** A significant increase in protein levels of HO1, NQO1, and TXNRD1 upon GTS21 (30 μm) treatment was noted using western blot in astrocytes treated with LPS for 24 h, and this effect was blocked by the 1 μm α7 nAchR antagonist MLA. *p < 0.05 compared with LPS 60 ng/ml treatment, ^#^ *p* < 0.05 compared with GTS21 30 μm + LPS 60 ng/ml treatment (both using one-way ANOVA with adjustment for multiple comparisons using Tukey’s test). Error bars represent SD (*n* = 3)

**Fig. 6:**
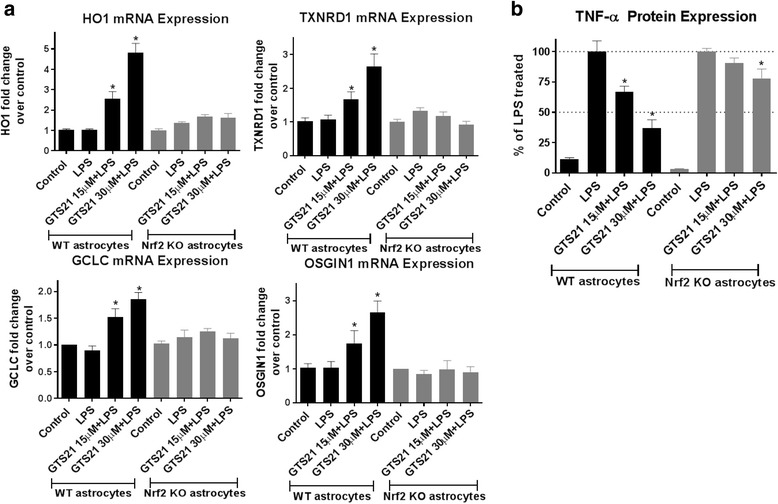
Antioxidant and anti-inflammatory responses of α7 nAchR activation are Nrf2 dependent. **a** Pretreatment with GTS21 (15 and 30 μm) resulted in significant upregulation of canonical Nrf2 antioxidant genes HO1, TXNRD1, GCLC, and OSGIN1 in wild-type astrocyte cultures treated with LPS (60 ng/ml) for 24 h, but not in astrocytes from Nrf2 knockout mice. *p < 0.05 compared with LPS (60 ng/ml) treatment (one-way ANOVA with adjustment for multiple comparisons using Dunnett’s test). Error bars represent SD (*n* = 4). **b** One hour pretreatment with GTS21 (15 and 30 μm) in astrocytes isolated from Nrf2 knockout mice treated with LPS (60 ng/ml) for 24 h resulted in substantially reduced blockage of pro-inflammatory cytokine, TNF-α, secretion (~ 22%) as compared with astrocytes cultured from wild-type mice (~ 63%). *p < 0.05 compared with LPS (60 ng/ml) treatment (one-way ANOVA with adjustment for multiple comparisons using Dunnett’s test). Error bars represent SD (*n* = 4)

We measured the expression profiles of a number of canonical Nrf2-responsive antioxidant genes. Pretreatment with GTS21 for 1 h resulted in a significant upregulation of HO1, TXNRD1, and GCLC in astrocytes treated with LPS for 24 h. This effect was significantly inhibited in the presence of α7 nAChR antagonist MLA (Fig. 5a). We also measured protein levels of Nrf2 target genes and found significant increase in HO1, TXNRD1, and NQO1, but no change in GCLC with GTS21 pretreatment in LPS-treated astrocytes (Fig. 5b). Further, a complete loss of this antioxidant response with GTS21 was observed in astrocytes cultured from Nrf2 knockout mice, suggesting that these effects are mediated through Nrf2 signaling (Fig. 6a).

### Astrocytes lacking Nrf2 have a reduced anti-inflammatory response to α7 nAChR activation

We evaluated the anti-inflammatory effects of GTS21 in astrocytes from Nrf2 knockout mice. Cell-free conditioned medium was collected from astrocytes cultured from either wild-type or Nrf2 knockout mice and treated with LPS for 24 h in the presence or absence of GTS21. As observed in the earlier experiments, GTS21 significantly reduced LPS-mediated TNF-α production in wild-type astrocytes. However, in Nrf2 knockout astrocytes, this effect was robustly blocked. These results indicate that activation of the Nrf2 pathway may in part be responsible for the anti-inflammatory effects of GTS21 (Fig. 6b).

### α7 nAChR activation inhibits neuronal apoptosis mediated by inflammatory astrocytes

**Fig. 7:**
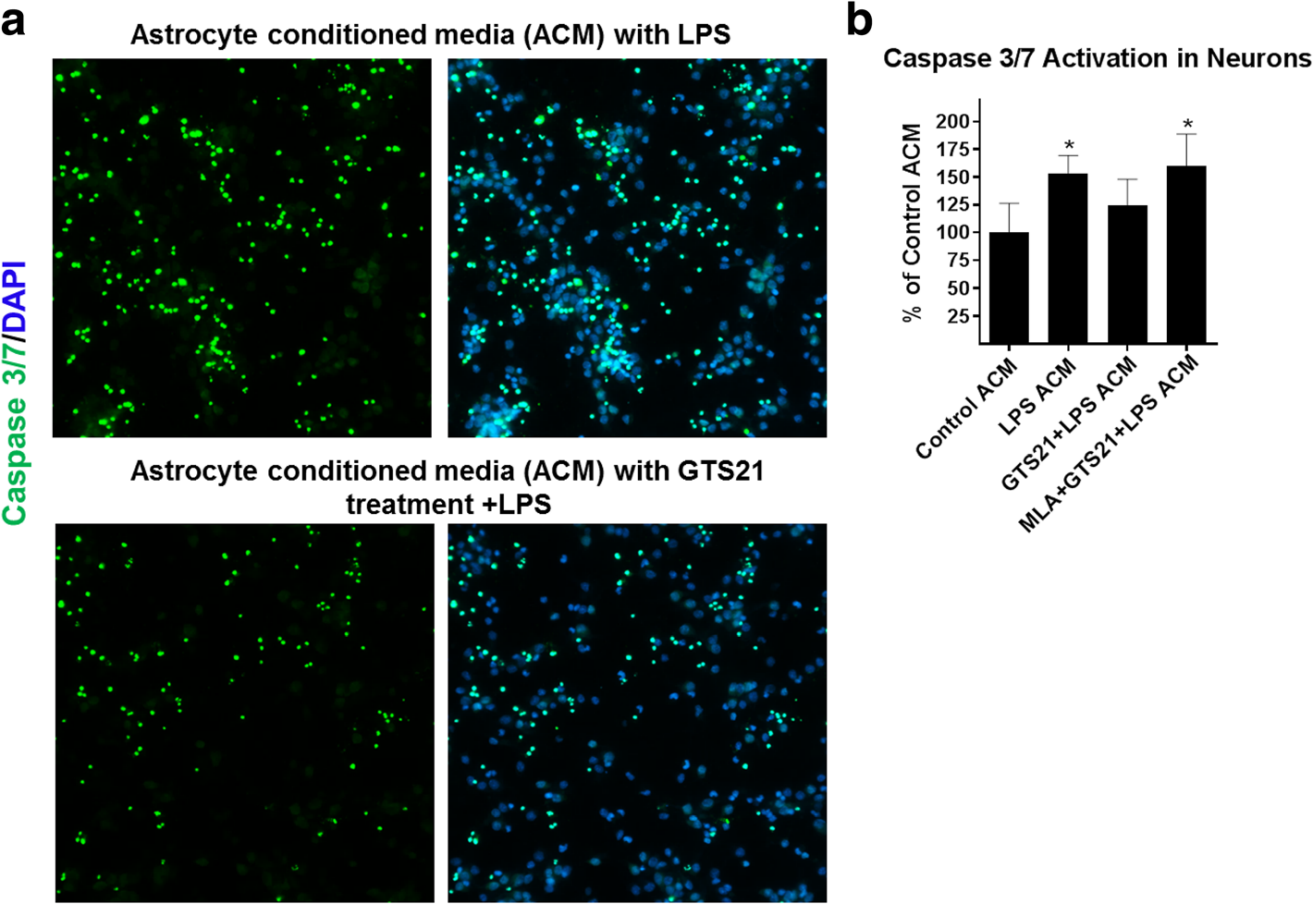
α7 nAchR activation reduced inflammatory astrocyte-mediated neuronal apoptosis. **a** Representative images of caspase activation in neurons treated with astrocyte conditioned media in the presence of LPS (60 ng/ml) for 24 h with or without 1 h GTS21 pretreatment (30 μm). **b** Quantification of caspase activation upon GTS21 (30 μm) pretreatment for 1 h in LPS (60 ng/ml)-activated astrocyte conditioned media showed significantly lowered levels of caspase 3/7 in neuronal cultures, and this effect was significantly blocked upon addition of 1 μm MLA, *p < 0.05 compared with control astrocyte conditioned media. *p < 0.05 compared with control (one-way ANOVA with adjustment for multiple comparisons using Dunnett’s test). Error bars represent SD (*n* = 5 wells, 15 fields per well)

Purified astrocytes were treated with LPS in the presence or absence of α7 nAchR agonist GTS21, and the cell-free conditioned medium was collected for addition to neurons. Significant apoptosis of neurons was observed in the presence of LPS-treated astrocyte conditioned medium. GTS21 pretreated astrocyte conditioned medium resulted in substantially reduced caspase activation suggesting less apoptosis. This effect was reversed by the α7 nAchR antagonist MLA, indicating the specificity of the response (Fig. 7a, b).

### α7 nAChR activation reduces NF-κB luciferase signal and pro-inflammatory genes downstream of the NF-κB signaling pathway in brain

**Fig. 8:**
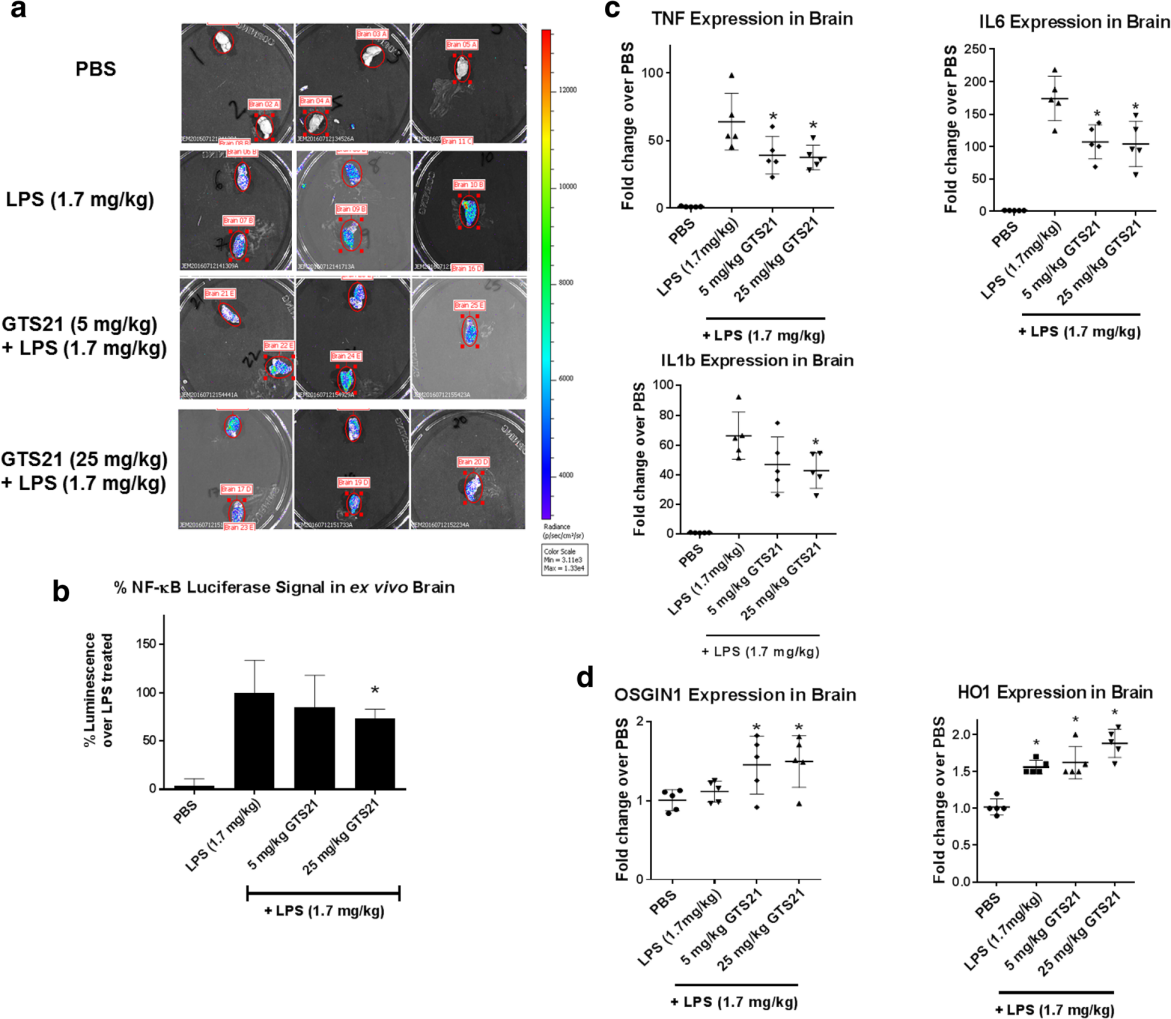
α7 nAchR activation reduced NF-κB luciferase signal and downregulated pro-inflammatory genes downstream of NF-κB signaling pathway in brain. **a** Representative images of NF-κB-dependent luminescence in one hemisphere of the brains (*n* = 5 mice per group) acquired on the IVIS Spectrum instrument (Perkin Elmer, Hopkinton, MA), using the Perkin Elmer proprietary software, Living Image (v4.3.1). Luciferin was injected intraperitoneally at 150 mg/kg for ex vivo brain imaging, which was conducted after euthanizing the animal and harvesting the brain. **b** NF-κB-dependent luminescence in the brains of NF-κB luciferase reporter mice was measured by imaging. A significant increase in NF-κB luciferase signal was observed with LPS (1.7 mg/kg) treatment, which was reduced in the presence of GTS21 (25 mg/kg) treatment. *p < 0.05 compared with LPS treated (one-way ANOVA with adjustment for multiple comparisons using Dunnett’s test). Error bars represent SD for *n* = 10 mice/group from two independent experiments. **c** RNA analysis of the other brain hemisphere from NF-κB luciferase reporter mice treated with LPS demonstrated statistically significant reductions in gene expression of inflammatory cytokines, TNF-α and IL-6 with 5 mg/kg and 25 mg/kg GTS21 treatment and IL-1β with 25 mg/kg GTS21 treatment, *p < 0.05 compared with LPS treatment (one-way ANOVA with adjustment for multiple comparisons using Dunnett’s test). Error bars represent SD for *n* = 5 mice/group. **d** Expression of OSGIN1 and HO1, which are downstream antioxidant genes of Nrf2 pathway, was significantly increased with GTS21 treatment, *p < 0.05 compared with PBS treatment (one-way ANOVA with adjustment for multiple comparisons using Dunnett’s test). Error bars represent SD for *n* = 5 mice/group

To study the effect of α7 nAChR activation on LPS-induced NF-κB luciferase signal in vivo, we used transgenic reporter mice where luciferase expression is driven by NF-κB activation. In the presence of luciferin, this expression results in bioluminescence, which was imaged and quantified. NF-κB activation was induced with LPS treatment. At 4 h of LPS treatment, there was a significant increase in NF-κB luciferase signal in the brain as compared to the PBS-treated control group. Animals injected with 25 mg/kg GTS21 demonstrated a reduction in the LPS-mediated NF-κB luciferase signal in the brains compared to LPS only treated animals (Fig. 8a, b).

After imaging, the other hemisphere of the brain was collected for RNA analysis of inflammatory cytokine downstream of the NF-κB signaling pathway. In mice treated with GTS21, significant reductions in gene expression of inflammatory cytokines TNF-α, IL-1β, and IL-6 were observed in the brain as compared to LPS only treated animals (Fig. 8c). We also measured the expression of the genes involved in the Nrf2 signaling pathway and observed increased expression of two Nrf2 target genes with GTS21 treatment in the brain as compared to the PBS-treated group: HO1 and oxidative stress-induced growth inhibitor (OSGIN1), which is activated early in the process of Nrf2 activation in the brain [25]. However, there was no significant difference in HO1 and OSGIN1 expression between LPS-treated group and LPS+GTS21-treated group (Fig. 8d). Collectively, these results demonstrate that activation of α7 nAChRs results in inhibition of the NF-κB pathway and upregulation of antioxidant Nrf2 target genes in an animal model of neuroinflammation.

## Discussion

In this study, treatment of astrocytes with α7 nAChR agonist GTS21 [26] reduced LPS-mediated inflammatory cytokines in a dose-dependent manner and this effect was reversed by both pharmacological and genetic inhibition of α7 nAChR expression. Further, α7 nAChR activation blocked LPS-mediated NF-κB nuclear translocation in astrocytes indicating that the observed anti-inflammatory effect may be mediated through the NF-κB pathway. We also demonstrated that treatment with α7 nAChR agonist upregulated canonical Nrf2 antioxidant genes and proteins suggesting antioxidant properties of α7 nAchR. Using an astrocyte conditioned media approach, we demonstrated a reduction in neuronal apoptosis with GTS21 treatment. Finally, in an in vivo neuroinflammation model using LPS in NF-κB luciferase reporter mice, we demonstrated reduction in LPS-induced NF-κB activity in the brains of animals treated with GTS21 using bioluminescent imaging. We also observed a reduction in the expression of pro-inflammatory cytokine downstream of the NF-κB pathway and an increase in Nrf2 target genes in brain tissues with GTS21 treatment.

α7 nAChRs have been recognized as a target with therapeutic relevance in neurodegenerative diseases because of their neuroprotective effects and potential association with cognitive enhancement [27, 28]. A distinct feature of these receptors is that they are widely expressed in neuronal and non-neuronal cells, including astrocytes, which are emerging as important players in neuroinflammation and cognitive impairment [17, 18]. However, the specific role of astroglial α7 nAChRs in neuroprotection has only been evaluated in a couple of prior investigations from a single laboratory. In one study, Liu et al. demonstrated that activation of astroglial α7 nAChRs may provide protection against degeneration of dopaminergic neurons by inhibition of MPTP (in vivo)- and MPP+- or LPS (in vitro)-induced astrocyte activation in PD [12]. In a second study, Liu et al. demonstrated that activation of α7 nAChRs resulted in a reduction in H_2_O_2_-induced apoptosis of astrocytes and expression of glial cell-derived neurotrophic factor [29]. Both of these studies point towards a critical role of astrocytes in neuroprotection conferred by α7 nAChR activation. The results from our study provide additional evidence for anti-inflammatory and antioxidant effects of astroglial α7 nAChR activation.

Further, our study also provides an explanation of the molecular mechanism of action for the observed anti-inflammatory effects of astroglial α7 nAChR activation. In LPS-treated astrocyte cultures from Nrf2 knockout mice, we not only observed a robust reduction of the antioxidant response of α7 nAchR activation but also the anti-inflammatory response in the in vitro inflammation model; suggesting that the Nrf2 and NF-κB pathways may work in concert to mediate the observed anti-inflammatory response of astroglial α7 nAchRs. Previous investigations have proposed multiple mechanisms by which the Nrf2 and NF-κB pathways may interact. First, the activation of the Nrf2 pathway leads to dissociation of Nrf2-Keap1 complex and increased free Kelch-like ECH-associated protein 1 (Keap1) in the cytosol. Keap1 is reported to bind with IKKβ, which may in turn prevent the phosphorylation of IκBα and p65 NF-κB subunit nuclear translocation leading to diminished NF-κB signaling [30]. On the other hand, Keap1 is also reported to be capable of binding directly with the p65 subunit of NF-κB [31]. Therefore, upon activation of the NF-κB pathway, this interaction may result in higher nuclear translocation of Keap1 and consequent functional inactivation of Nrf2, in turn exaggerating the inflammatory response. Another potential interaction between the two pathways involves CREB-binding protein (CBP), which is a transcription co-activator capable of binding to both Nrf2 and the phosphorylated p65 subunit of NF-κB [32]. Since Nrf2 and NF-κB compete for CBP, knocking out Nrf2 results in higher binding of NF-κB/p65 to CBP and consequently higher transcription of inflammatory genes, leading to a more potent inflammatory response. Our observation of decreased anti-inflammatory response of α7 nAChR activation in astrocytes from Nrf2 knockout mice is consistent with this hypothesis.

The Nrf2 pathway has received increasing recognition as a major player in glial α7 nAChRs-mediated neuroprotection. Parada et al. reported that cell death and ROS production were reduced upon treatment with α7 nAChR agonist PNU282987 in organotypic hippocampal slice cultures under oxygen and glucose deprivation conditions, and these effects were reduced substantially upon immunotoxic depletion of microglial cells [33]. In vivo, these significantly diminished antioxidant and neuroprotective effects were noted upon α7 nAChR activation in HO1 knockout mice, suggesting active involvement of the Nrf2 pathway in neuroprotection against brain ischemia. Findings from our study confirm these observations regarding the importance of the Nrf2 pathway in glial α7 nAChR-mediated neuroprotection. Aditionally, our study critically notes that the Nrf2 pathway is not only involved in α7 nAChR-mediated antioxidant effects, but may also interact with the NF-κB pathway to reduce pro-inflammatory cytokine secretion.

Activation of functional α7 nAChRs expressed on astrocytes has been demonstrated to produce rapid currents and increase intracellular calcium levels [11]. After an initial influx of calcium through astroglial α7 nAChR channels, further modulation of calcium signaling occurs through calcium-induced calcium release from intracellular store [34]. Increase in intracellular calcium in astrocytes is reported to regulate synaptic transmission and plasticity [35, 36]. Astrocytes reside in close proximity to neurons and are key components of neural circuits. Recent evidence suggests that calcium signaling cascades in astrocytes result in increased extracellular glutamate levels, thereby shifting the local neural circuit to a slow oscilation state of synchronized neuronal firing critical in memory consolidation and sleep [37]. Taken together, these studies suggest that activation of astroglial α7 nAChRs may play a role in enhancement of cognitive function. Our results showing anti-inflammatory and antioxidant properties of astroglical α7 nAChRs suggest that in pathological states such as neurodegenerative diseases, targeting these receptors can provide additional benefits through neuroprotection.

## Conclusion

In conclusion, our results suggest that activating astroglial α7 nAChRs may have a role in neuroprotection by decreasing inflammation and oxidative stress, and therefore could have therapeutic implications for the development of disease modifying treatments for neurodegenerative diseases. The anti-inflammatory effects observed with activation of astroglial α7 nAChRs appear to be mediated through an interaction of the Nrf2 and NF-κB pathways. Our findings of robust anti-inflammatory and antioxidant effects of astroglial α7 nAChR stimulation provide a compelling rationale for future research evaluating this response in specific neurodegenerative disease models to guide development of novel therapeutics targeting these receptors.

## Abbreviations

CBP: CREB-binding protein
CNS: Central nervous system
GAPDH: Glyceraldehyde-3-phosphate dehydrogenase
GCLC: Glutamate-cysteine ligase catalytic subunit
GFAP: Glial fibrillary acidic protein
HO1: Heme oxygenase 1
I κB: Nuclear factor of kappa light polypeptide gene enhancer in B cell inhibitor, alpha
IL: Interleukin
Keap1: Kelch-like ECH-associated protein 1
LPS: Lipopolysaccharide
MPP+: 1-Methyl-4-phenylpyridinium ion
MPTP: 1-Methyl-4-phenyl-1,2,3,6-tetrahydropyridine
nAChRs: α7 nicotinic acetylcholine receptors
NF-κB: Nuclear factor kappa-light-chain-enhancer of activated B cells
NO: Nitric oxide
NO_2_^–^: Nitrite
NQO1: NAD(P)H:quinone oxidoreductase-1
Nrf2: Nuclear factor erythroid-derived 2-related factor 2
OSGIN1: Oxidative stress-induced growth inhibitor 1
ROS: Reactive oxygen species
TNF-α: Tumor necrosis factor alpha
TXNRD1: Thioredoxin reductase 1
